# Evaluating Thyroid Lesions Using Fine-Needle Aspiration Cytology and the Bethesda System: Insights From a Tertiary Care Hospital

**DOI:** 10.7759/cureus.98277

**Published:** 2025-12-01

**Authors:** Nikhil Kumar, Nishi, Nidhi Priya Barla, Ranwir Sinha, Rahul Kumar Bharti, Prima Lakra, Monalisa Katyare, Dheeraj Kumar, Sanjeet Singh

**Affiliations:** 1 Pathology, All India Institute of Medical Sciences, Deoghar, Deoghar, IND; 2 Pathology and Laboratory Medicine, All India Institute of Medical Sciences, Deoghar, Deoghar, IND; 3 Pathology and Laboratory Medicine, All India Institute of Medical Sciences, Bhopal, Bhopal, IND; 4 Otolaryngology - Head and Neck Surgery, All India Institute of Medical Sciences, Deoghar, Deoghar, IND

**Keywords:** bethesda system of reporting thyroid cytopathology, colloid goiter, fine needle aspiration cytology (fnac), fna, thyroid cytology

## Abstract

Introduction

Thyroid enlargement is a common clinical condition often evaluated by various modalities, but fine-needle aspiration cytology (FNAC) remains the gold standard for accurate diagnosis and surgical decision-making. This study aimed to assess the cytological spectrum of thyroid lesions and evaluate the impact of FNAC in reducing unnecessary surgical interventions in a tertiary care center.

Materials and methods

A combined prospective and retrospective study was conducted on 282 patients with thyroid lesions at the All India Institute of Medical Sciences, Deoghar, Deoghar, Jharkhand, India, from August 2023 to March 2025. FNAC samples were classified according to the 2023 Bethesda System for Reporting Thyroid Cytopathology (TBSRTC). Clinical and cytomorphological data were analyzed to determine lesion distribution and malignancy risk.

Results

The majority of cases (85.1%) were benign (Bethesda category II), with colloid goiter (42.55%) and lymphocytic thyroiditis (28.01%) being most prevalent. Indeterminate (category III) and suspicious/malignant lesions (categories IV-VI) collectively accounted for approximately 15% of cases. No non-diagnostic samples were reported, attributed to on-site adequacy evaluation and ultrasound-guided aspirations. A significant association was observed between gender and cytological diagnosis (p=0.010), with lymphocytic thyroiditis being more common in females and suspicious papillary carcinoma more frequent in males. Confirmed papillary carcinoma constituted 1.8% of cases, comparable to previous studies. The Bethesda System effectively stratified malignancy risk, guiding appropriate clinical management.

Conclusion

FNAC is a cost-effective, minimally invasive tool that reliably distinguishes benign from malignant thyroid lesions, thereby reducing unnecessary surgeries. The Bethesda System standardizes reporting and enhances communication between clinicians and pathologists, supporting evidence-based management. Ultrasound guidance and strict adherence to adequacy criteria optimizes diagnostic yield, making FNAC indispensable in thyroid lesion evaluation.

## Introduction

Thyroid enlargement is one of the most frequently encountered clinical conditions in routine medical practice [1]. Although various diagnostic modalities, such as physical examination, ultrasound (USG), and thyroid scintigraphy, are commonly employed to evaluate thyroid abnormalities, they often lack the specificity and sensitivity required for definitive diagnosis [2]. In contrast, fine-needle aspiration (FNA) has gained widespread acceptance as a first-line diagnostic tool due to its simplicity, safety, cost-effectiveness, and diagnostic reliability. USG-guided FNA plays an important role in thyroid cytopathology. It plays a critical role in the evaluation of thyroid nodules by distinguishing lesions that require surgical management from those that can be managed conservatively [3].

Fine-needle aspiration cytology (FNAC), in particular, is regarded as the most accurate and reliable method for determining the need for surgical intervention in patients with thyroid nodules [4]. It has significantly influenced clinical decision-making by reducing unnecessary surgeries for benign lesions, thereby improving patient outcomes and optimizing resource utilization.

The aim of the present study was to evaluate the cytological spectrum of thyroid lesions in a tertiary care center. By analyzing cytomorphological features, the study intends to categorize thyroid lesions based on standardized reporting criteria.

## Materials and methods

Study design and setting

This was a prospective and retrospective observational study conducted in the Department of Pathology, All India Institute of Medical Sciences (AIIMS), Deoghar, Deoghar, Jharkhand, India, over a period of 20 months (August 2023 to March 2025). The study included all patients who presented with thyroid swellings and underwent FNAC for diagnostic evaluation during the study period.

The study protocol was reviewed and approved by the Institutional Ethics Committee (IEC) of AIIMS Deoghar. Written informed consent was obtained from all patients prior to the procedure for participation and use of their clinical data for research purposes.

Study population

The study included patients who were admitted to inpatient wards or referred from any clinical and surgical outpatient department to the Department of Pathology for cytological evaluation.

Inclusion criteria

All patients presenting with thyroid swellings or nodules, irrespective of age, gender, or clinical presentation, were included in the study.

Exclusion criteria

Patients with non-thyroidal neck swellings or lesions not arising from the thyroid gland and who did not provide informed consent were excluded from the study.

Sample size

A total of 282 patients fulfilling the inclusion criteria were enrolled in the study. Each case represented a unique FNAC sample from an individual patient.

Clinical evaluation and data collection

Detailed clinical information was obtained for all cases, including demographic details, duration and location of swelling, and associated symptoms such as pain, dysphagia, dyspnea, or hoarseness. Relevant clinical history included prior thyroid disease, radiation exposure, or a family history of thyroid disorders.

Clinical examination findings, thyroid function tests (T3, T4, TSH), and ultrasonographic features (where available) were also recorded.

Fine-needle aspiration procedure

FNAC was performed using standard aseptic precautions in the cytopathology section of the Department of Pathology. A 22-24-gauge needle attached to a 10-mL disposable syringe was used. Multiple passes were made in different directions within the lesion to ensure adequate sampling. The aspirated material was carefully expressed onto clean glass slides.

Air-dried smears were prepared and stained with May-Grünwald-Giemsa (MGG) stain. Alcohol-fixed smears (95% methanol) were stained using the Papanicolaou (PAP) method for optimal nuclear detail. In cases with cystic lesions, both fluid and residual solid components were aspirated and processed separately to ensure cellular adequacy.

Cytomorphological evaluation

All stained smears were examined independently by two experienced cytopathologists using a light microscope under low and high magnifications. Smears were assessed for cellularity and colloid amount, architecture (microfollicular, macrofollicular, or dispersed pattern), nuclear features (enlargement, grooves, inclusions, chromatin pattern), the presence of oncocytic cells, lymphocytes, multinucleated giant cells, and colloid characteristics. Any discordant findings were reviewed jointly to arrive at a final consensus diagnosis.

Diagnostic categorization

All cases were categorized according to The Bethesda System for Reporting Thyroid Cytopathology (TBSRTC), 2023 revision, which classifies thyroid FNAC findings into six diagnostic categories [4,5] (Table 1). This system is widely accepted for reporting thyroid cytopathology.

**Table 1:** The Bethesda System for Reporting Thyroid Cytopathology (TBSRTC) 2023 Source: [5]

Category	Diagnosis
I	Non-diagnostic
II	Benign
III	Atypia of undetermined significance (AUS)
IV	Follicular neoplasm
V	Suspicious for malignancy (SFM)
VI	Malignant

Statistical analysis

All data were compiled in Microsoft Excel and analyzed using SPSS Version 25.0 (IBM Corp., Armonk, NY). Descriptive statistics were used to calculate frequencies, percentages, and mean ± standard deviation (SD) for continuous variables. The distribution of cases across Bethesda categories, age groups, and gender was analyzed, and correlations between cytological diagnosis and clinical parameters were evaluated where applicable.

## Results

A total of 282 cases that met the inclusion criteria were included in this study. The majority of thyroid lesion cases were observed in the age group of 20-39 years (47.52%), followed by the age group of 40-59 years (34.4%), indicating a higher prevalence among middle-aged adults (Table 2).

**Table 2:** Age-wise distribution of thyroid lesion cases in the study population

Age group (years)	Number of cases	Percentage
11-19	27	9.57
20-39	134	47.52
40-59	97	34.40
>60	24	8.51
Total	282	100

The study population showed a marked female predominance (87.23%), with a female-to-male ratio of approximately 6.8:1 (Table 3).

**Table 3:** Sex distribution of the study population

Sex	Number of cases	Percentage (%)
Female	246	87.23
Male	36	12.77
Total	282	100

Among the 282 cases analyzed (Table 4), colloid goiter was the most common diagnosis, observed in 120 (42.55%) cases. This was followed by lymphocytic thyroiditis, seen in 79 (28.01%) cases, indicating a high prevalence of benign inflammatory lesions. Other variants of colloid goiter included cases with cystic changes (6.38%) and those associated with lymphocytic thyroiditis (2.13%).

**Table 4:** Spectrum of thyroid lesions in the study population

Diagnosis	Number of cases	Percentage (%)
Colloid goiter	120	42.55
Colloid goiter with cystic changes	18	6.38
Colloid goiter with lymphocytic thyroiditis	6	2.13
Lymphocytic thyroiditis	79	28.01
De Quervain thyroiditis	1	0.4
Follicular nodular disease	11	3.90
Hyperplastic nodule	2	0.71
Hashitoxicosis	2	0.71
Atypia of undetermined significance	13	4.61
Follicular neoplasm	7	2.5
Suspicious of papillary carcinoma	15	4.6
Suspicious of medullary carcinoma	2	0.7
Papillary carcinoma	5	1.8
Anaplastic carcinoma	1	0.4
Total	282	100.0

Less common benign conditions included follicular nodular disease (3.90%), hyperplastic nodule (0.71%), hashitoxicosis (0.71%), and De Quervain thyroiditis (0.4%). Among the indeterminate and neoplastic categories, atypia of undetermined significance (AUS) was noted in 13 (4.61%) cases, and follicular neoplasm in 7 (2.5%) cases. Suspicious and malignant diagnoses were relatively less frequent. Suspicious for papillary carcinoma accounted for 15 (4.6%) cases, suspicious for medullary carcinoma for 2 (0.7%) cases, while confirmed papillary carcinoma and anaplastic carcinoma were diagnosed in 5 (1.8%) cases and 1 (0.4%) case, respectively.

This distribution highlights that the majority of thyroid lesions in the study were benign, with a smaller but notable proportion requiring further evaluation or surgical management due to suspicion or confirmation of malignancy.

The majority of thyroid cytology cases in the study were classified under Bethesda category II, accounting for 85.10% (240 cases), indicating that most lesions were benign. Category III (AUS) comprised 4.60% (13 cases), suggesting a small proportion of indeterminate results requiring further evaluation. Category V (suspicious for malignancy) accounted for 6.03% (17 cases), while categories IV (follicular neoplasm) and VI (malignant) each contributed 2.13% (Table 5).

**Table 5:** Case distribution according to The Bethesda System for Reporting Thyroid Cytopathology (TBSRTC)

Category	Number of cases	Percentage (%)
Category I	0	0
Category II	240	85.10
Category III	13	4.61
Category IV	6	2.13
Category V	17	6.03
Category VI	6	2.13
Total	282	100.0

Malignant cases (category V) included papillary carcinoma and anaplastic carcinoma. This distribution reflects a predominance of benign lesions in the study population, with a smaller proportion showing indeterminate or suspicious/malignant features.

Due to the implementation of on-site evaluation during FNAC procedures at our institution, non-diagnostic samples were identified and repeated immediately at the time of aspiration. This approach ensured that all reported cases met the adequacy criteria as per TBSRTC. Another reason for the absence of non-diagnostic cases is that the procedure was performed by a cytopathologist, with ultrasound-guided FNA undertaken whenever required.

The present data highlights the distribution of thyroid cytological diagnoses across different age groups and genders (Table 6). While no statistically significant association was observed between age groups and the types of thyroid lesions (p = 0.32), gender showed a significant correlation with diagnostic category (p = 0.010).

**Table 6:** Association of age and gender with various cytology-based diagnoses of thyroid lesions (N=282) #P-value based on the chi-square test. *Statistically significant (p<0.05). df, degrees of freedom

Characteristics	Colloid goiter (N=120), n (%)	Colloid goiter with cystic change (N=18), n (%)	Lymphocytic thyroiditis (N=79), n (%)	Atypia of underdetermined significance (N=13), n (%)	Suspicious papillary carcinoma (N=15), n (%)	Others (N=37), n (%)	Chi-square value, df	p-Value^#^
Age group (years)								
11–19	12 (44.4)	0	8 (29.6)	1 (3.7)	2 (7.4)	4 (14.8)	17.1, 15	0.32
20–39	63 (47.0)	10 (7.5)	38 (28.4)	2 (1.5)	5 (3.7%)	16 (11.9)		
40–59	35 (36.1)	7 (7.2)	30 (30.9)	8 (8.2)	6 (6.2)	11 (11.3)		
≥60	10 (41.7)	1 (4.2)	3 (12.5)	2 (8.3)	2 (8.3)	6 (25.0)		
Gender								
Male	18 (50.0)	3 (8.3)	2 (5.6)	3 (8.3)	5 (13.9)	5 (13.9)	15.2, 5	0.010*
Female	102 (41.5)	15 (6.1)	77 (31.3)	10 (4.1)	10 (4.1)	32 (13.0)		

**Figure 1:**
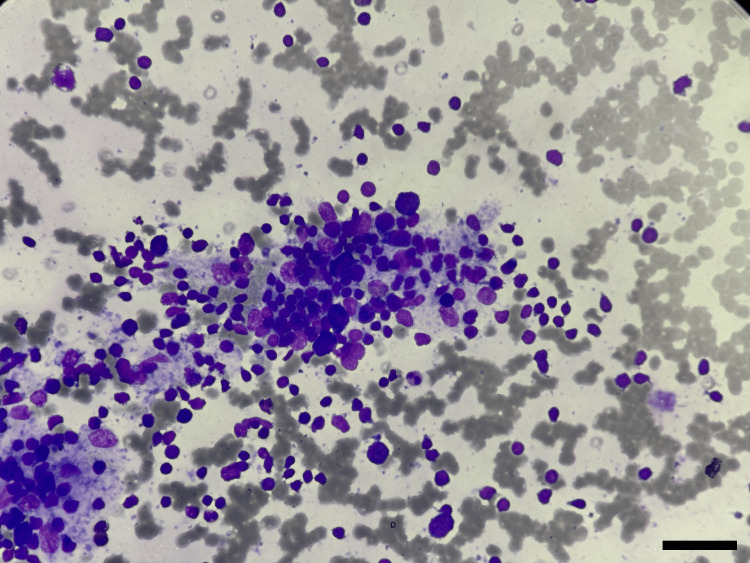
Cytological smear of lymphocytic thyroiditis (category II) Cytological smear showing lymphocytic impingement on follicular cells (Giemsa stain; x400)

Colloid goiter was the most common diagnosis overall, particularly among individuals aged 20-39 years. Lymphocytic thyroiditis was predominantly seen in females, aligning with the known autoimmune predisposition in women (Figure 1).

**Figure 2:**
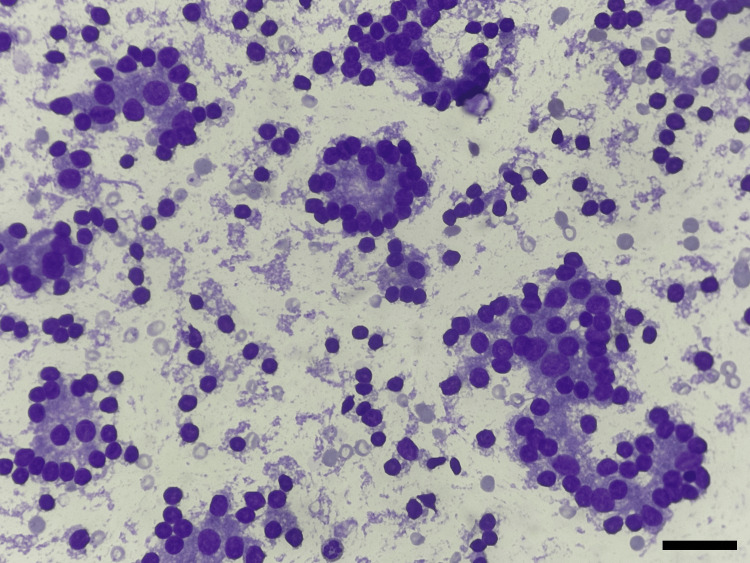
Cytological smear showing follicular cells arranged in microfollicles (category IV) Smear showing follicular cells arranged in repetitive follicles (Giemsa stain, ×400)

Follicular neoplasm was also noted in around 2.13% of cases (Figure 2).

**Figure 3:**
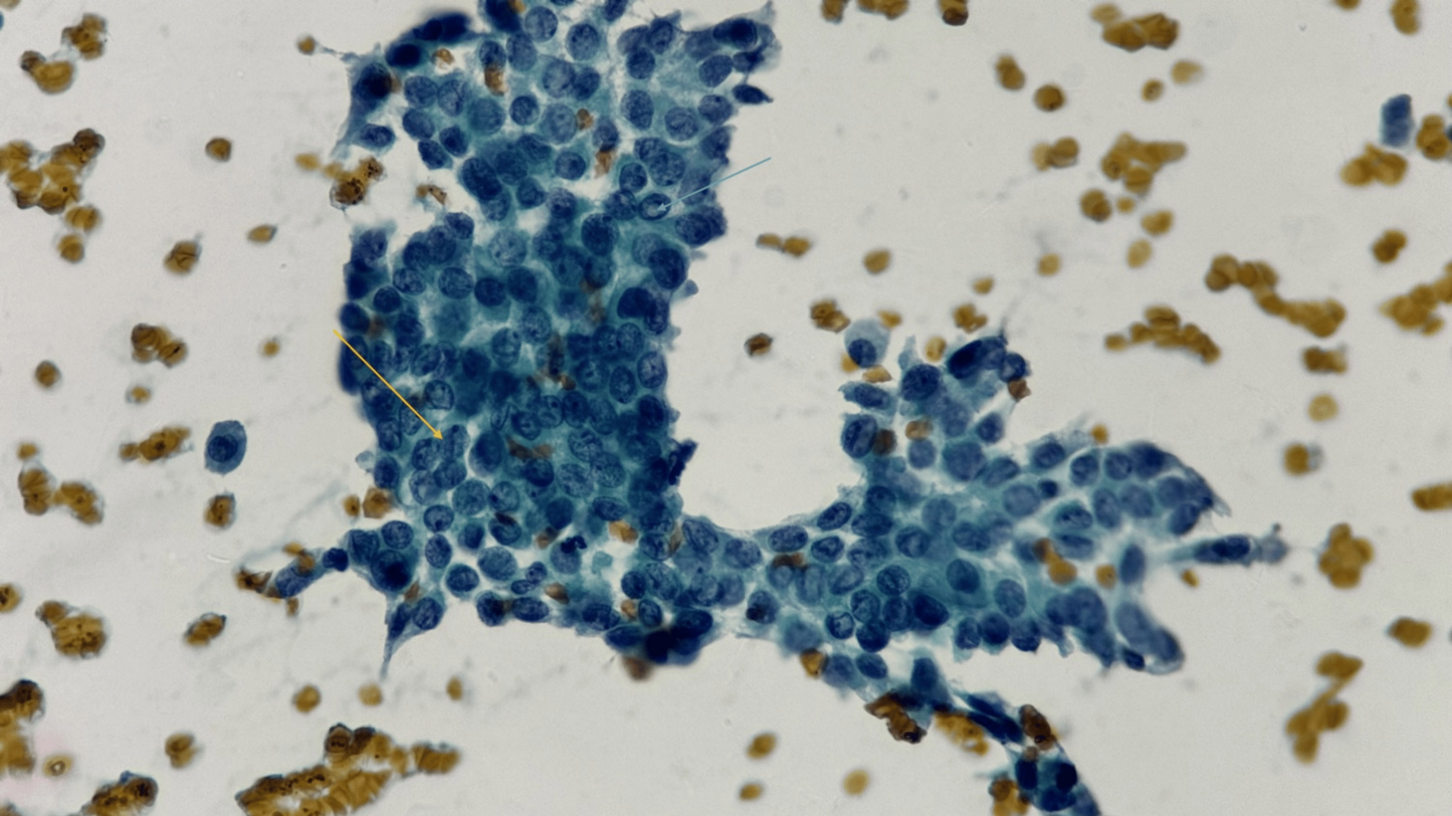
Cytological smear of a suspicious papillary carcinoma Cytological smear showing cohesive clusters with overlapping, enlarged nuclei, powdery chromatin, and nuclear grooves (Yellow arrow). Occasional intranuclear cytoplasmic inclusions (blue arrow) are noted. The background shows scant colloid material (Papanicolaou stain, ×400; scale bar = 20 µm).

In contrast, a higher proportion of males were diagnosed with suspicious papillary carcinoma (Figure 3), suggesting a potential gender disparity in the presentation of more serious thyroid pathology.

**Figure 4:**
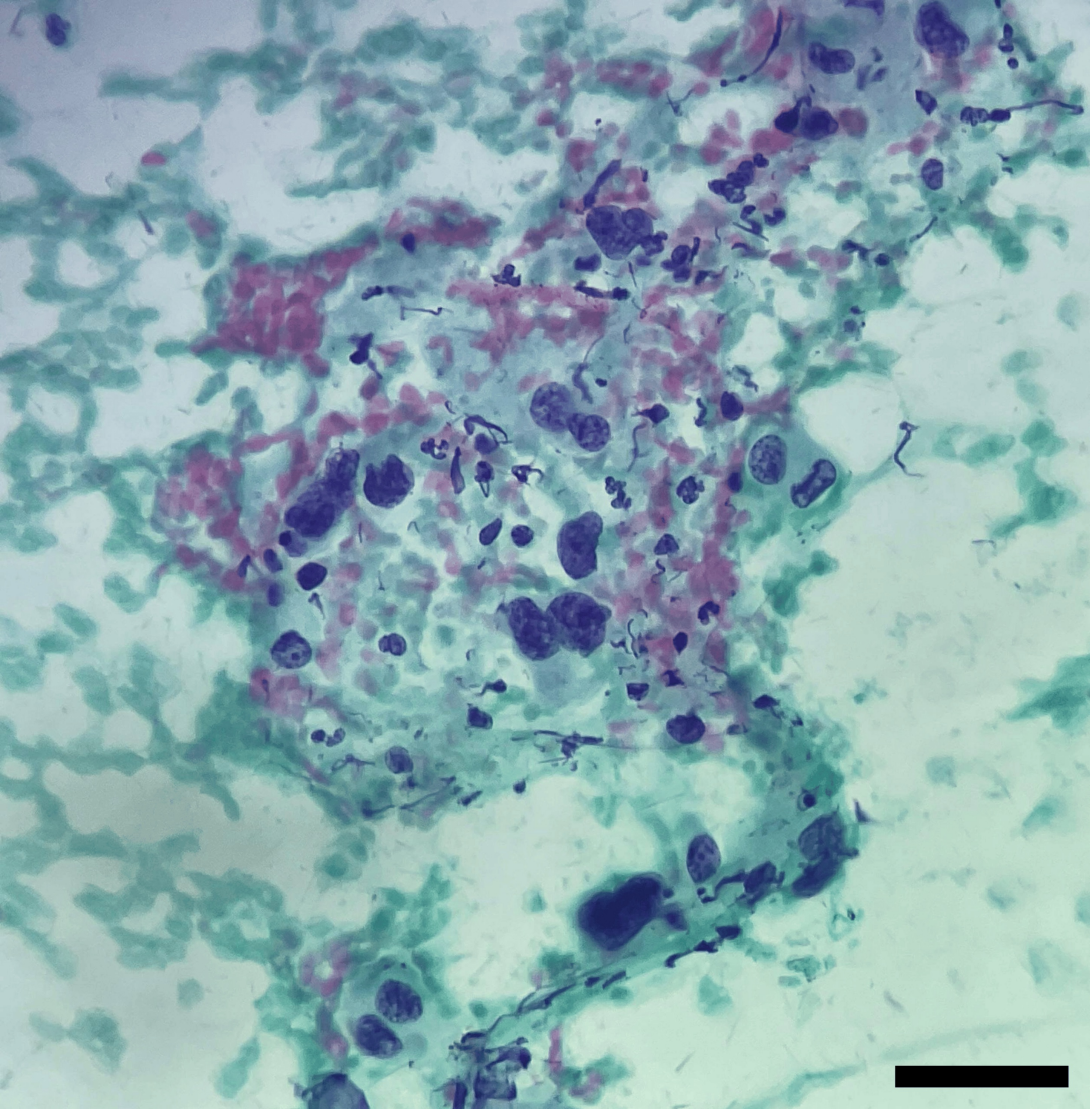
Cytological smear of anaplastic carcinoma Cytological smear showing highly pleomorphic tumor cells with marked nuclear atypia, irregular nuclear membranes, coarse chromatin, and prominent nucleoli, consistent with anaplastic carcinoma (category VI) (Papanicolaou stain, ×400; scale bar = 20 µm).

Malignant cases (category V) included papillary carcinoma and anaplastic carcinoma. Anaplastic carcinoma usually occurs in the older age group with the presence of very atypical cells (Figure 4).

## Discussion

This study analyzed 282 thyroid cytology cases, revealing a clear predominance of benign lesions, with colloid goiter and lymphocytic thyroiditis forming the majority of diagnoses. These findings are consistent with previous research, reinforcing the typical distribution pattern of thyroid lesions commonly encountered in cytological evaluations.

These findings collectively reaffirm the utility of FNAC as the first-line diagnostic tool in the evaluation of thyroid nodules. Its simplicity, accuracy, and cost-effectiveness make it indispensable in routine clinical practice.

Most cases in our study were observed in the age group of 20-39 years, indicating that thyroid lesions frequently affect individuals during their most active and productive phase of life. This trend closely mirrors the observations of Hathila et al., who reported a peak incidence in the age group of 31-50 years [6].

A marked female predominance was evident, reaffirming the well-established female susceptibility to thyroid disorders. This observation aligns with the results of Nautiyal et al., who documented a male-to-female ratio of 1:6.14 [7]. Hormonal and autoimmune influences, particularly the immunomodulatory effects of estrogen, likely contribute to this striking disparity, underscoring the gender-linked predisposition to thyroid pathology.

Lind et al. in their study emphasized on applying USG-guided FNAC for better diagnostic sensitivity of FNAC. In the present study, USG-guided FNA was performed whenever required [8].

All cases were categorized according to the 2023 TBSRTC (3rd edition), which continues the six-tier framework - Non-diagnostic, Benign, AUS, FN, SFM, and Malignant - while incorporating refinements that enhance diagnostic precision and clinical decision-making [5].

In a study by Kanukuntla et al., the benign category was the largest (83.87%), followed by the malignant category (6.04%). Similarly, in our study, benign lesions predominated, followed by category V (suspicious for malignancy) [9].

Interestingly, no cases fell under category I (non-diagnostic). This may be attributed to the routine use of USG-guided FNAC for small or heterogeneous nodules and the direct involvement of cytopathologists during aspiration. As noted by Nandedkar et al., this approach significantly improves sample adequacy and minimizes non-diagnostic results [10]. In contrast, Ansari et al. and Bodh et al. reported non-diagnostic rates of 6.5% and 2.98%, respectively [11,12]. Departmental protocols emphasizing real-time adequacy assessment likely contributed to the absence of such cases in our study.

Colloid goiter was the most common diagnosis (42.55%), followed by lymphocytic thyroiditis (28.01%). Similar findings were reported by Agrawal et al. (62.68% colloid goiter, 10.44% lymphocytic thyroiditis) and Nandedkar et al. (57.41% colloid goitre), confirming that benign thyroid lesions form the bulk of FNAC diagnoses worldwide [2,10].

A strong female predominance (87.2%) was evident in our series, comparable to the findings of Patel and Patel, who reported a female-to-male ratio of 3.76:1 [13]. The significant association between gender and cytological diagnosis (p = 0.010) highlights this predisposition, particularly for lymphocytic thyroiditis, which was markedly higher among females in our cohort. This pattern aligns with the well-established autoimmune tendency in women, driven by hormonal and immunological factors [14]. Lymphocytic thyroiditis, therefore, stands as a classic example of how autoimmunity and gender biology intersect in thyroid pathology.

In contrast, age did not show a statistically significant association with cytological categories (p = 0.32), indicating that thyroid lesions can manifest across a wide age spectrum, without a specific age predilection.

The majority of cases were classified as Bethesda category II (benign) at 85.10%, in agreement with Patel and Patel, who reported a similar 85.9% in their cohort [13]. Categories III (AUS) and V (suspicious for malignancy) accounted for 4% and 3.2%, respectively. The AUS category, known for its diagnostic ambiguity, represented 4.61% of cases. This is well within the <10% threshold recommended by the TBSRTC [15].

Follicular neoplasm constituted 2.13% of cases, closely matching the 1.84% reported by Handa et al. and, papillary carcinoma was confirmed in 1.8% of cases, comparable to the 2.3% incidence [16]. The low prevalence of malignancy underscores the fact that most thyroid nodules are benign, highlighting FNAC as a reliable, cost-effective, and minimally invasive tool for early triage. By differentiating between benign and suspicious lesions, FNAC helps avoid unnecessary surgeries and supports conservative management in low-risk cases.

Overall, by providing risk estimates of malignancy for each diagnostic category, the Bethesda System continues to play a crucial role in guiding clinicians on follow-up strategies, surgical indications, and the extent of intervention required.

Moreover, the application of the updated Bethesda System (2023) ensures better risk stratification and consistency in reporting, bridging the gap between cytopathology and clinical decision-making. Continued efforts toward standardization, audit, and correlation with histopathology will further refine diagnostic precision and strengthen the role of FNAC as a cornerstone in thyroid lesion evaluation.

Limitations of the study

The study is limited by its lack of follow-up histopathological correlation for all cases, which would strengthen diagnostic accuracy assessments. Being a single-center study, the findings may not be generalizable to other populations or settings.

## Conclusions

FNAC is a cost-effective, minimally invasive procedure that provides a specific diagnosis with rapid turnaround and minimal complications. In cases with benign cytology findings, patients can often be safely managed with clinical and radiological follow-up, avoiding unnecessary surgery. TBSRTC offers a standardized and clinically meaningful framework for interpreting and reporting thyroid FNAC results. Its simplified, systematic approach enhances communication between cytopathologist and clinicians, leading to more consistent and evidence-based management strategies. Adopting the updated Bethesda System for reporting thyroid cytology ensures consistent interpretation, reduces diagnostic ambiguity, and promotes optimal patient care.
